# Exploring consumer perceived risk and purchase intention of water-saving appliances: A moderated dual-mediation model

**DOI:** 10.3389/fpsyg.2022.1099897

**Published:** 2023-01-10

**Authors:** Teng Wang, Ming Tian

**Affiliations:** ^1^Department of Management and Human Resources, Business School, Hohai University, Nanjing, China; ^2^Department of Marketing, Business School, Hohai University, Nanjing, China

**Keywords:** water-saving appliance, perceived risk, consumer trust, consumer knowledge, purchase intention

## Abstract

With the blooming of the socio-economy in China, urban water consumption continues rising, and the promotion of water-saving appliances has become one of the priorities of water saving efforts. Based on the perceived risk theory, this research constructs a moderated mediation model to explore the mechanisms that explain and affect consumers’ willingness to purchase water-saving appliances. The study finds that consumers’ perceived risk of buying water-saving appliances is mainly functional, economic, and psychological risks. Perceived risk will reduce consumers’ quality trust and green trust in water-saving appliances, and indirectly influences consumers’ willingness to buy through quality and green trust. In addition, we find that consumer knowledge of water-saving appliances can weaken the negative impact of perceived risk on quality trust and green trust and the indirect inhibitory effect on purchase intentions. In final, we provide policy recommendations to guide consumers to purchase water-saving appliances and promote the popularization of water-saving appliances.

## Introduction

In 2020, China’s national domestic water consumption was 86.31 billion m^3^, accounting for 14.9% of the total water consumption in that year (Ministry of Water Resources, 2021). With the rapid social and economic development in recent years, the water shortage has become increasingly prominent among water resource issues. In total freshwater consumption, household water consumption has become critical as the water demands from urban and rural residents rise continuously. Specifically, in 2020, Beijing’s household water consumption is 956 million m^3^, reaching 37.5% of the total production and domestic water consumption (Beijing Water Authority, 2021). Considering the increasing amount of household water consumption, the popularization of water-saving appliances has become important for improving water usage efficiency and establishing a water-saving society. In the *National Water-conservation Action Plan* jointly issued by the National Development and Reform Commission and the Ministry of Water Resources, the promotion of water-saving appliances has been clearly listed as one of the primary directions of water-conservation efforts in China. However, currently, the popularization of water-saving appliances in the consumer market in China is quite limited. For instance, the popularization rate of household water-saving appliances in 2019 is only 30% (Gou, 2019). Therefore, increasing consumers’ purchase intention of water-saving appliances has been challenging for promoting water conservation.

As Batchelor et al. (2014) stated, the promotion of water-saving appliances has been extensively identified as the best way to save water unconsciously. In extant wisdom, the purchase intention of water-saving appliances is considered a function of individual differences and product characteristics. For example, based on the Spanish consumers’ sample, Martínez-Espiñeira and García-Valiñas (2013) found that education and income levels positively impact the purchase intention of water-saving appliances, while age had a negative impact. Hustvedt et al. (2013) investigated the purchase intention of American consumers for water-saving washing machines and found that the energy-saving and water-saving performance, cost, and publicity channels of washing machines are the main factors affecting consumers’ purchase intention. Tapsuwan et al. (2018) found in the purchase intention of water-saving sinks for Sydney households that consumers’ purchase intention is affected by product function, use environment, and consumers’ personality characteristics. Mu et al. (2014) found that water-saving knowledge, the degree of new and old houses, family size, and actual water prices will affect consumers’ purchase of water-saving appliances. Fan et al. (2019) found that consumers’ education level, income level and water-saving knowledge, as well as the water-saving and energy-saving performance of washing machines, will significantly affect the purchase intention of water-saving washing machines.

Although these studies offer insightful ideas about the factors that influence consumers’ purchase intention of water-saving appliances, recent work has been limited in two important ways. Initially, those studies primarily focus on positive factors such as income level and water-saving knowledge, while mostly neglecting the negative impacts of consumers’ perceived risk on water-saving appliances. Water-saving appliances belong to technologically innovative products in terms of water efficiency, and consumers usually perceive various potential risks when purchasing innovative products (Yin et al., 2019). In product transactions, perceived risk reduces consumers’ expectations of the reliability of the seller or product (Hong and Cho, 2011). Regarding water-saving appliances, will perceived risks weaken consumers’ trust in water-saving appliances and the corresponding purchase intentions? Next, these studies assume that all consumers who perceived risk would act the same way in purchasing water-saving appliances and ignored variations among the difference in consumers. From the perspective of information asymmetry theory, mastering product knowledge can help consumers find internal information clues (Lee et al., 2015), which helps resist external uncertainties’ interference. Therefore, another question is how consumers’ knowledge about water-saving appliances shapes the impact of perceived risk on consumers’ trust and purchase intention.

To address the issues above, we draw on the theory of perceived risk and examine how and when consumers’ perceived risk influences their purchase intention of water-saving appliances. We explore the mediation mechanism of consumer trust, and the contingent conditional effect of consumers’ product knowledge of water-saving appliances. Perceived risk theory argues that consumers prefer to minimize their perceived risk (Mitchell, 1999; Chen and Chang, 2012). However, the information asymmetry makes consumers hard to identify actual product value before purchase (Park et al., 2016), which raises their risk perceptions and negative consumption emotions about purchasing the products. In this condition, risk-related emotions such as anxiety or worry would negatively affect trust (Eid, 2011), and eventually purchase intention (Chang and Chen, 2008). Besides, information processing differs between customers with high and low levels of product knowledge (Selnes and Howell, 1999). Consumers with a high level of product knowledge could search for intrinsic information cues and process information less based on emotional factors (Lee et al., 2015). Thus, when consumers occupy a high level of product knowledge, their trust and purchase intention are less susceptible to perceived risk.

Our research makes several contributions to the current literature. First, unlike previous studies that primarily focus on consumer demographic and product characteristics, we explored consumers’ perceived risk influencing the purchase intention of water-saving appliances. The research findings extend the psychological foundation of promoting consumer purchase intention on water-saving appliances. Then, regarding how perceived risk influences the purchase intention of green products, previous literature mainly focused on the mediation effect of green trust (Chen and Chang, 2012; Juliana et al., 2020), whereas it neglected the dual mediation effect of green trust and quality trust. By examining the dual mediation effect of quality trust and green trust, our study provides a more comprehensive understanding of the mechanism that transmits perceived risk into purchase intention. Lastly, we examine how consumer knowledge moderates the influence of perceived risk on consumer trust and, subsequently, purchase intention. Our findings revealed a nuanced process of how perceived risk interacts with consumer knowledge to shape consumers’ trust and purchase intention of green products such as water-saving appliances.

The remainder of this paper is organized as follows. Following the Introduction, “Theory and hypotheses” briefly reviews the related literature and develops the research hypotheses. The research model is presented in “Materials and methods,” and “Hypotheses testing” analyzes the data and tests hypotheses. “Discussion and implications” discusses the research results and provides theoretical and policy implications. In the final Section, we highlight the limitations and future research directions.

## Theory and hypotheses

### Consumers’ perceived risks of purchasing water-saving appliances

The concept of perceived risk was originally proposed by Bauer (1967), who defined perceived risk as “the combination of uncertainty and severity of the outcome involved.” Because this concept is highly context-dependent, it is usually defined based on the research setting in the following literature. For instance, in consumers’ selection of online payment tools, perceived risk is defined as “the potential loss in pursuit of the expected outcome of using electronic services” (Yang et al., 2015). In public acceptance of nuclear energy technology, Wang et al. (2019) define perceived risk as “the degree to which individuals perceive themselves to be at risk (such as nuclear leakage and radiation) when developing and utilizing nuclear energy.” Hwang and Choe (2020) defined perceived risk as “the subjective expectation of loss from visiting edible insect restaurants” in consumption intentions of edible insect restaurants.

In urban domestic water consumption, water-saving appliances refer to “devices or appliances that incorporate water-saving technology, can reduce water flow, water consumption, and improve water efficiency” (Ministry of Housing and Urban-Rural Development, 2014). Specifically, water-saving appliances usually include faucets, showers, toilets, washing machines, dishwashers, etc. In China, the popularization of water-saving appliances is still in the start-up stage, and the technology research and development and market promotion are immature. Consumers could face various uncertainties and perceive risks when purchasing water-saving appliances (Huang, 2017). Combined with the previous wisdom and the situation water-saving appliance market in China, we define the perceived risk of water-saving appliance purchases as “consumers’ subjective expectations of potential losses in purchasing water-saving appliances.”

The types of perceived risk can be traced back to the classic six-category framework proposed by Cunningham, including performance/function risk, financial/economic risk, opportunity/time risk, safety/physical risk, social risk, and psychological risk (Cunningham, 1967). Follow-up research divides consumers’ perceived risk types according to the research background and settings. In a study of online payment tool choice, Yang et al. (2015) argue that consumers’ perceived risks include economic, functional, security, time, privacy, service, and psychological risks. In the research on consume intention of edible insect restaurants, Hwang and Choe (2020) divided perceived risk into seven types: quality, psychology, health, finance, environment, time, and society. In the research on online travel booking, Park et al. (2016) divided perceived risks into eight types: time, economy, function, privacy, security, psychological, physical, and equipment. Chen et al. (2019) divided consumers’ perceived risks into four types: financial, physical, time, and function in studying the purchase intention of new energy vehicles.

Based on the relevant literature and water-saving appliances’ product characteristics, we argue that consumers could perceive four types of risk when purchasing water-saving appliances: function, economy, psychology, and time. Functional risk refers to consumers’ perception of uncertainty in product performance, especially the possible defects of water-saving appliances in terms of performance, design, and compatibility compared to traditional water-saving appliances. Economic risk refers to the economic loss caused by purchasing, repairing, and maintaining water-saving appliances. Psychological risk refers to the potential pessimistic impact on consumers’ inner state or self-perception when purchasing water-saving appliances. Time risk refers to the risk that water-saving appliances will cause consumers to spend extra time on learning, installing, and daily usage.

Theoretically, the overall perceived risk is indicated by the second-order factor consisting of multiple risk facets, such as function, economic, psychological risk, etc. (Park et al., 2016). Previous studies have found that the second-order model of perceived risk is more efficacious in comparison with the first-order model (Park et al., 2016; Marriott and Williams, 2018). The overall risk could well reflect the influence of each perceived risk facet; hence it is unnecessary to add each risk facet to the list for predicting the influence of overall perceived risk (Martins et al., 2014). As such, in the context of water-saving appliances, we follow the previous wisdom and model perceived risk as a second-order composite variable.

### The impact of perceived risk on purchase intention

As a subjective perception of the negative consequences and uncertainties of post-consumption, consumers’ assessment of perceived risk affects their purchasing decisions. According to the theory of perceived risk, consumers have the tendency to minimize risk (Mitchell, 1999), so an increase in perceived risk will decrease consumers’ willingness to buy. Extant literature has confirmed the negative impact of consumers’ perceived risk on purchase intention, such as purchasing new-energy vehicles and the food delivery based on mobile terminals (Liu and Zhao, 2021). Due to the inherent information asymmetry in the transaction, it is difficult for consumers to judge the actual value of a product when purchasing (Mishra et al., 1998), thus inhibiting consumers’ willingness to purchase. Specifically, when consumers perceive purchasing a product as unacceptably risky, they are generally less likely to purchase it.

In China, water-saving appliances are immature in the consumer market, and most consumers cannot fully understand the actual value of water-saving appliances. Due to the imperfection of national standards, industry standards, and market permit institutions, water-saving appliances’ quality varies considerably (Huang, 2017). In addition, water-saving appliances are more expensive than regular water appliances in purchasing and maintaining. In terms of time cost, consumers need to spend extra time learning and installing water-saving appliances. Finally, water-saving appliances may also negatively influence consumers’ psychological states. The problems in product quality, price, and time costs may individually or jointly cause consumers anxiety when purchasing and using water-saving appliances.

In sum, the uncertainties mentioned above could increase consumers’ perceived risk of purchasing water-saving appliances. Under the influence of consumers’ general risk aversion characteristics, consumers’ willingness to purchase water-saving appliances is ultimately inhibited. Based on the above, the following assumptions are put forward:

*H1*: Consumers’ perceived risk negatively impacts their purchase intention of water-saving appliances.

### The mediating role of consumer trust

Mayer et al. (1995) believe that “trust is the expectation of a party to perform its specific obligations and the willingness to accept possible damages in a transaction, regardless of its ability to control the other party.” In the research on the purchase intention of organic cotton, consumer trust refers to “a sense of security that consumers believe that the purchased product can meet their consumption expectations” (Tong and Su, 2018). Referring to the above literature, we define consumer trust in purchasing water-saving appliances as “the psychological state of consumers who believe that water-saving appliances can meet their consumption expectations.”

In the context of water-saving appliances, consumers’ trust in water-saving appliances includes two facets: quality trust and green trust, and their consumption expectations are twofold, the cleaning function and the water efficiency, respectively. We define *quality trust* as “consumers’ trust in the cleaning functions of water-saving appliances, “such as the shower experience and the clean effect of washing machines and dishwashers. Besides, consumers have consumption expectations for environmental performance when purchasing green products (Chen and Chang, 2012), unlike the trust in the quality of generic products or services such as online payment (Yang et al., 2015) or organic products (Tong and Su, 2018). Following Chen’s (2010) definition of the general green trust as “a willingness to depend on one object based on the belief or expectation resulting from its credibility, benevolence, and ability about environmental performance,” we define consumers’ *green trust* in water-saving appliances as “consumers’ expectations for water-saving performance,” such as water efficiency and water-saving performance.

In purchase decision-making, the formation of a trusting belief could be based on the level of perceived risk (Yang et al., 2015). When facing an unfamiliar product, information asymmetry makes consumers hard to identify the actual product value before purchase (Park et al., 2016), which raises their risk perception and correlated negative emotions. Under such circumstances, risk-related emotions such as anxiety or worry would negatively affect trust (Eid, 2011). As a result, consumers who perceive risks tend to show a low trust level. For instance, Zhang et al. (2019) found that perceived risk can significantly reduce consumers’ trust in autonomous vehicles. Similarly, consumers would perceive various risks in terms of function, economy, psychology, and time when purchasing water-saving appliances. Therefore, they have doubts and worries about the cleaning functions and water-saving capabilities of the appliances, which ultimately reduces consumers’ quality trust and green trust. Based on the above, the following hypotheses are put forward:

*H2a*: Consumers’ perceived risk negatively affects quality trust in water-saving appliances.

*H2b*: Consumers’ perceived risk negatively affects green trust in water-saving appliances.

Consumer trust is essential for companies to obtain consumers’ purchasing intentions. Studies such as online shopping (Zhang et al., 2021), and organic food purchases (Yu et al., 2021) have verified the positive impact of consumer trust on purchase intention. As consumers trust increases, anxiety and uncertainty are reduced, and the integrity of the brand or company is strengthened (Chen et al., 2015). Regarding purchasing water-saving appliances, consumer trust is the psychological state of thinking that water-saving appliances can meet consumer expectations regarding cleaning ability and water-saving efficiency. Consumers believe that water-saving appliances can meet the expectation of product quality, user experience, and water-saving effects, thereby generating purchase intention. Therefore, the following assumptions are put forward:

*H3a*: Consumers’ quality trust positively affects purchase intention of water-saving appliances.

*H3b*: Consumers’ green trust positively affects purchase intention of water-saving appliances.

Combining the above arguments, the mediation effect is likely to hold true. In the context of water-saving appliances, the relationship between perceived risk and purchase intention is likely indirect and mediated by consumer’s quality trust and green trust in water-saving appliances. The perceived risk will influence quality trust and green trust, and subsequently, purchase intention.

In terms of consumers’ trust in functional performance, a strong perception of product risk will result in a low level of quality trust in water-saving appliances. Perceived risk in a product purchase refers to the uncertainty regarding the outcome and the associated expectation of losses. It could inhibit consumer trust and purchase behavior (Hong and Cho, 2011). If a product purchase is considered risky, the customer will show more negative emotions regarding this purchase (Wang and Hazen, 2016). Those negative emotions will compromise consumers’ trust in the functional performance of water-saving appliances, especially when they doubt that water efficiency may have a trade-off with cleaning power. In this circumstance, a consumer would be cautious about the usage quality when considering purchasing water-saving appliances. Could low-flow toilets flush waste as well as their standard counterparts? Is the water-efficiency washing machine reliable and durable? Will the showerhead save water by sacrificing the expected shower experience? If the consumer doubts functional performance and durability, he or she will put little quality trust in the merchant, thereby leading to a low purchase intention.

Besides, a consumer could have doubts about the environmental performance of the water-saving appliances, which potentially impairs their green trust in the products. Since consumer purchase intentions are positively affected by consumer trust (Harris and Goode, 2010), the decreased green trust will lower the purchase intention. For example, consumers considering the purchase of water-saving products would be most likely serious about water efficiency, whereas this sort of environmental performance is usually difficult to perceive before purchasing (Chen and Chang, 2012). In a recent survey on the adoption of water-efficient washing machines in China, water efficiency has been identified as one of the main factors contributing to purchase intention (Fan et al., 2019). In this condition, a consumer considering the purchase of a water-saving washing machine would usually be concerned if the water efficiency is worth the price. If customers doubt that the water efficiency cannot meet their expectations, they will begin to distrust the water-saving appliances, and then probably avoid purchasing.

The above arguments have indicated the mediating role of consumers’ quality trust and green trust in the relationship between perceived risk and purchase intention. Perceived risk in water-saving appliances will reduce consumers’ green and quality trust, ultimately weakening their willingness to purchase. Hence, the following hypotheses are put forward:

*H4a*: Quality trust mediates the relationship between consumers’ perceived risk and purchase intention of water-saving appliances.

*H4b*: Green trust mediates the relationship between consumers’ perceived risk and purchase intention of water-saving appliances.

### The moderating role of consumer knowledge of water-saving appliances

Product knowledge refers to “a consumer’s awareness of specific information concerning a given product” (Wang and Hazen, 2016). In the purchase decision-making of products or technologies, consumer product-related knowledge plays an important role. Consumers often use their product-related knowledge to evaluate products and tend to purchase based on their knowledge relevant to the products. What a consumer knows about a product is crucial in the case of information-intensive products (Vigar-Ellis et al., 2015). Water-saving products are such information-intensive products that are often conveyed in a highly specialist format, requiring consumers’ environmental knowledge to enable information processing. As suggested by Thøgersen et al. (2012), consumers’ reactions of environmental products may vary across different consumer knowledge levels. Thus, in this current study, we anticipated that consumers’ green trust and quality trust toward water-saving appliances with perceived risk would differ based on their level of knowledge. More specifically, perceived risk has a stronger effect on trust among low-knowledge consumers than their high-knowledge counterparts.

In the relevant literature, consumer knowledge has long been examined as one of the boundary conditions that can change outcomes. High-knowledge consumers can better search for more information and develop a more comprehensive understanding of decision-making (Kumar et al., 2021). They, therefore, rely more on cognitive judgments rather than on other cues (King and Balasubramanian, 1994). When evaluating water-saving products, consumers with high knowledge will rely on existing knowledge and cognitive evaluation and will be less likely to be influenced by perceived risk. Thus, compared with low-knowledge consumers, their trust in water-saving appliances is more likely to hold when they perceive risks. In contrast, consumers with low knowledge need more background knowledge to process product information. Hence their trust in water-saving appliances could be more compromised by cues that imply risk associated with the products.

Furthermore, in the context of product purchasing, perceived risk is composed of the individual’s subjective feelings of certainty that the outcome will be unpleasant (Lee, 2009). The risk-related unpleasant emotions are crucial factors that can reduce consumer trust in purchasing products (Chen and Chang, 2012). Under this circumstance, consumers with high knowledge are more confident and less confused (Mazursky and Vinitzky, 2005). They are less likely to be influenced by emotional states when determining their final attitudes toward products (Lee, 2016), because their decisions are based more on their product-related knowledge (Devlin, 2011). In the context of water-saving appliances, consumers with higher knowledge could be less vulnerable to risk-related emotions, and their trust would be less susceptible to perceived risks. In other words, for consumers with a relatively high product knowledge, their trust in water-saving appliances is less negatively affected by perceived risk. Based on the above, the following hypotheses are put forward:

*H5a*: The relationship between perceived risk and quality trust is moderated by consumer knowledge, such that the relationship is weaker for consumers with a higher knowledge of water-saving appliances.

*H5b*: The relationship between perceived risk and green trust is moderated by consumer knowledge, such that the relationship is weaker for consumers with a higher knowledge of water-saving appliances.

**Figure 1:**
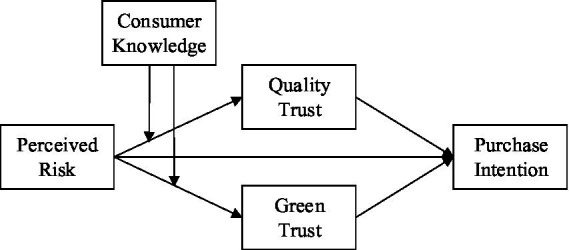
Theoretical model.

As our arguments above stated, consumer knowledge moderates the relation between perceived risk and consumer trust, and consumer trust is positively associated with purchase intention. We further propose that consumer knowledge moderates the mediating effects of consumer quality trust and green trust in the relation between perceived risk and purchase intention—a moderated dual-mediation model (Figure 1).

Consumers’ product knowledge is consistently considered as one of the moderators of the relationship between consumers’ perception and purchase intention (Fu and Elliott, 2013). In the context of water-saving appliances, when consumers have a lower level of product knowledge, the negative influence of perceived risk would be strengthened, thereby reducing consumer trust and, subsequently, purchase intention. In contrast, when consumers have a higher level of product knowledge, the negative influence of perceived risk on trust would be weakened, thereby the negative impact of risk on purchase intention *via* trust would be mitigated. Alternatively, compared with a lower level of product knowledge, a higher level of product knowledge would weaken the indirect effect of consumer quality trust and green trust in the relation between perceived risk and purchase intention. Based on the above, the following assumptions are put forward:

*H6a*: The indirect influence of perceived risk on purchase intention via quality trust will be weaker for consumers with a high level of knowledge.

*H6b*: The indirect influence of perceived risk on purchase intention via green trust will be weaker for consumers with a high level of knowledge.

## Materials and methods

### Sample and procedures

Our questionnaire consists of five parts. To begin, we introduce the definition and scope of water-saving appliances and ask respondents to answer whether they have experience purchasing water-saving appliances, the channels for learning about water-saving appliances, and their knowledge of water-saving appliances. Next, we ask respondents to answer questions about perceived risk when purchasing water-saving appliances, including functional risk, economic risk, psychological risk, and time risk (Oliver, 1997; Park et al., 2016). Then, the third part measures consumers’ quality and green trust in water-saving appliances. The following fourth part measures consumers’ willingness to purchase water-saving appliances. Finally, we collect demographic information on gender, age, education level, job position, income level, and regions. Based on the pre-test of 137 valid questionnaires, the final questionnaire includes 21 core items and 6 demographic items. To control the common method bias caused by the self-assessment questionnaire, we follow Newton et al. (2015) and arrange the measurement items of the independent and dependent variables appeared in a non-sequential manner.

In the data collection, we conducted an online survey and recruited participants using WeChat, a multipurpose mobile application for messaging, social media, and mobile payments (Westjohn et al., 2022). The snowball sampling method was employed (Yu et al., 2020). We first invited participants from different provincial administrative regions to answer the questionnaire. Meanwhile, they were asked to spread the survey to their family, friends, or colleagues. Finally, 448 questionnaires were returned. Following the questionnaire review procedure employed in the previous literature (Wang et al., 2019), we discarded the samples with missing values and screened participants based on a long string greater 6 (DeSimone et al., 2015). In addition, we also threw out samples based on a response time of less than 3 min. Finally, we obtained 337 valid questionnaires, and the effective response rate was 75.22%. Among the 337 valid questionnaires, 231 were female (68.55%), and 106 were male (31.45%); In terms of age, 272 were under 30 years old (81.90%), 45 were 31–45 years old (13.35%), and over 46 years old 16 people (4.75%). For the education level, 117 respondents with graduate degrees and above (34.72%), 213 respondents with bachelor’s degree (63.20%), and 7 respondents with high school and technical secondary school or below (2.08%).

For the profession, there are 187 students (55.49%), 109 enterprises and government employees (32.34%), and 41 others (12.17%). In terms of monthly income, 214 people were below 5,000 Chinese yuan (63.50%), 71 people were 5,000–10,000 yuan (21.07%), 34 people were 10,000–15,000 yuan (10.09%), and 18 people were more than 15,000 yuan (5.34%). Regarding regions, 163 people come from water-deficient areas (48.37%), and 174 people from non-water-deficient areas (51.63%).

### Measure

In our study, all the scales were developed based on previous research. Following the translation and back-translation procedure (Brislin, 1970), the items were obtained from the previous literature, then translated (English to Chinese) and back-translated (Chinese to English) by two bilingual scholars to ensure the validity of the translation in a cross-cultural setting. One scholar is a marketing professor in the United States, and the other scholar is a management professor at a university in China. All measures were rated using the Likert 7-point scale, ranging from strongly disagree (1) to strongly agree (7). Table 1 reports the detailed content of items.

**Table 1:** Reliability and validity test results.

Main construct and items	Loadings	CR	Cronbach’s *α*	AVE
Function Risk, FR		0.856	0.854	0.664
Compared with traditional water appliances, the function and quality of water-saving appliances are still not ideal	0.791			
Water-saving appliances have limited functionality and will be difficult to meet my needs	0.809			
Water-saving appliances have poor user experience due to design or performance issues	0.844			
Economic Risk, ER		0.844	0.837	0.644
Compared with traditional water appliances, the maintenance cost of water-saving appliances is higher	0.789			
Maintenance and other costs make the total cost of water-saving appliances higher than I expected	0.885			
The water bills saved by the water-saving appliances hardly make up for the extra costs they incur	0.726			
Psychological Risk, PR		0.920	0.917	0.794
Buying water-saving appliances creates additional worries for me compared to traditional water appliances	0.809			
Compared to traditional water appliances, buying water-saving appliances will make me feel less psychologically relaxed	0.948			
Buying water-saving appliances creates unwanted anxiety in me compared to traditional water appliances	0.910			
Consumer Knowledge, CK		0.900	0.898	0.751
I am very knowledgeable about water saving appliances	0.900			
I am familiar with some common water saving appliances	0.842			
I have received a lot of information about water saving appliances	0.856			
Quality Trust, QT		0.861	0.860	0.673
I’m willing to buy water saving appliances despite the possible risks	0.825			
I think water saving appliances are reliable and trustworthy	0.829			
I believe water saving appliances can meet my usage needs	0.807			
Green Trust, GT		0.877	0.876	0.704
I believe in the water saving performance of water saving appliances	0.826			
I believe that water saving appliances can effectively save water resources	0.868			
I believe water economizers are beneficial for improving water efficiency	0.823			
Purchase Intention, PI		0.858	0.857	0.669
Generally speaking, I am willing to buy water saving appliances	0.878			
I choose to buy water saving appliances if possible	0.815			
I want to buy despite the uncertainty of the water saver	0.756			

Perceived risk was measured using the scale from Yang et al. (2015) and Park et al. (2016). Perceived risk is a second-order factor that consists of functional risk, economic risk, and psychological risk. The scale of consumer knowledge was developed by Wang et al. (2019), and we employed it to measure consumers’ understanding of water-saving appliances. We measure quality trust by the scale developed by Oliveira et al. (2017), which mainly measures consumers’ trust in the quality of water-saving appliances. Meanwhile, we measure green trust by the scale developed by Chen and Chang (2012), which measures consumers’ belief in the water-efficiency capability of water-saving appliances. We assessed purchase intention using the scale developed by Yang et al. (2015). The control variables were selected based on previous consumer purchase intention literature (Tong and Su, 2018), including gender, age, education, occupation, income, and region.

### Model test

The second-order factor setting of the perceived risk construct was tested using STATA 17.0. In the exploratory factor analysis, the eigenvalue of the time risk was less than 1, and all the fit indicators dropped after including time risk in CFA. Therefore, consumer perceived risk is a second-order construct composed of three first-order factors: functional risk, economic risk, and psychological risk.

We use confirmatory factor analysis (CFA) to assess the rationality of the model set. We examine the discriminant validity and convergent validity by indicators such as Cronbach’s alpha, average variance extraction (AVE), and construct reliability (CR). In Table 2, CFA results suggest that the model included all the constructs fit the best (*χ*^2^/*df* = 2.331, CFI = 0.946, TLI = 0.935, SRMR = 0.060, RMSEA = 0.063). Table 1 reports the reliability and validity of each construct. All the items significantly loaded on the corresponding construct; factor loadings range from 0.756 to 0.948. The Cronbach’s *α* and combined reliability (CR) of each construct are greater than 0.8, and the average extraction variance (AVE) is all greater than 0.6, significantly better than the acceptable critical value (Hair et al., 1998). In Table 3, AVE’s square root is larger than the correlation coefficient between constructs (Fornell and Larcker, 1981), indicating good discriminant validity for the constructs.

**Table 2:** Model fit and indicators.

Model fit indicators	*χ*^2^/*df*	CFI	TLI	SRMR	RMSEA
Criterion	< 3	> 0.9	> 0.9	< 0.08	< 0.1
CFA model (2nd order without time risk)	2.331	0.946	0.935	0.060	0.063
CFA model (2nd order with time risk)	2.432	0.931	0.923	0.071	0.070
CFA model (1st order)	2.931	0.927	0.913	0.092	0.076
SEM model	1.725	0.959	0.952	0.067	0.046

**Table 3:** Descriptive statistics, correlation and average extraction variance (AVE).

	1	2	3	4	5	6	7	8
Perceived risk	***0.837***							
Function risk	0.800^***^	***0.815***						
Economic risk	0.755^***^	0.464^***^	***0.802***					
Psychologic risk	0.807^***^	0.435^***^	0.400^***^	***0.891***				
knowledge	0.216^***^	0.085	0.109^**^	0.295^***^	***0.867***			
Quality trust	−0.151^***^	−0.142^***^	−0.062	−0.144^***^	0.272^***^	***0.820***		
Green trust	−0.128^**^	−0.132^**^	0.002	−0.153^***^	0.224^***^	0.681^***^	***0.839***	
Purchase intention	−0.157^***^	−0.108^**^	−0.022	−0.217^***^	0.249^***^	0.716^***^	0.650^***^	***0.818***
Messan	4.250	4.163	4.865	3.722	3.852	5.318	5.556	5.533
S.D.	1.086	1.376	1.201	1.551	1.527	0.978	0.983	0.994

*N* = 337, ^*^p < 0.1, ^**^p < 0.05; ^***^p < 0.01; the bold diagonal text is the square root of AVE.

We test the model of “risk perception - > quality/green trust - > purchase intention” to examine the influence path. The results show that the SEM model fits well (*χ*^2^/*df* = 1.73, CFI = 0.959, TLI = 0.952, SRMR = 0.067, RMSEA = 0.046; see Table 2). We employed Harman’s single factor test for the common variance bias; the percentage of explained variance for the first common factor was 29.37% and far less than the 50% critical value (Liu and hao, 2021). Therefore, there is no obvious common method bias issue that could seriously impact the analysis results.

## Hypotheses testing

### Regression analysis

To test the hypotheses above, we conducted hierarchical multiple regression analysis by entering control variables, independent variable (perceived risk), and mediator variables (green trust and quality trust) in separate steps. In line with previous studies on the purchase intention of green products, we controlled for social-demographic factors such as gender, education, age, occupation, and income of respondents. Besides, considering that water scarcity could influence an individual’s concern about water-saving (Mahafza et al., 2017), we also control whether the respondent is located in water-shortage provinces.

Table 4 reports the regression analysis results. The results show that (1) perceived risk is negatively related to quality trust in water-saving appliances (*β* = −0.197, *p* < 0.01, Model 1); (2) The interaction between perceived risk and consumer knowledge is positively related to quality trust (*β* = 0.094, *p* < 0.01, Model 2); (3) perceived risk is negatively related to green trust on water-saving appliances (*β* = −0.150, *p* < 0.01, Model 3); (4) The interaction between perceived risk and consumer knowledge is positively related to green trust (*β* = 0.065, *p* < 0.05, Model 4); (5) Perceived risk is negatively related to purchase intention on water-saving appliances (*β* = −0.184, *p* < 0.01, Model 5); (6) The effect of perceived risk on purchase intention is not significant (*β* = −0.039, *n.s*, Model 6) when quality trust (*β* = 0.515, *p* < 0.01, Model 6) and green trust (*β* = 0.285, *p* < 0.01, Model 6) both present. In addition, the adjusted *R*-squared varies from 0.123 in model 1–0.564 in model 6, indicating that the explanatory power of the dual mediation model is the most robust.

**Table 4:** Results of hierarchical regression analysis.

	Quality trust		Green trust		Purchase intention	
	Model 1	Model 2	Model 3	Model 4	Model 5	Model 6
Perceived risk	−0.197^***^	−0.221^***^	−0.150^***^	−0.166^***^	−0.184^***^	−0.039
	(−4.546)	(−4.955)	(−3.366)	(−3.581)	(−4.032)	(−1.083)
Perceived risk × knowledge		0.094^***^		0.065^**^		
		(3.442)		(2.359)		
Quality trust						0.515^***^
						(8.717)
Green trust						0.285^***^
						(5.267)
Gender	0.101	0.092	−0.218^*^	−0.224^*^	−0.137	−0.127
	−0.800	−0.735	(−1.756)	(−1.800)	(−1.070)	(−1.338)
Education	−0.086	−0.080	−0.058	−0.054	0.010	0.070
	(−0.843)	(−0.786)	(−0.580)	(−0.538)	(0.090)	(0.989)
Age	0.229^**^	0.215^**^	0.147	0.137	0.103	−0.056
	(2.440)	(2.349)	(1.501)	(1.438)	(1.023)	(−0.742)
Occupation	0.008	0.013	0.063	0.066	0.025	0.003
	(0.145)	(0.235)	(1.077)	(1.125)	(0.393)	(0.071)
Income	−0.007	−0.030	0.107^*^	0.091	0.058	0.031
	(−0.103)	(−0.481)	(1.708)	(1.434)	(1.058)	(0.847)
Region	0.062	0.039	0.068	0.052	−0.097	−0.148^**^
	(0.598)	(0.380)	(0.634)	(0.489)	(−0.921)	(−2.031)
Knowledge	0.188^***^	0.189^***^	0.173^***^	0.174^***^	0.204^***^	0.058^*^
	(4.856)	(4.939)	(4.838)	(4.807)	(5.096)	(1.869)
_cons	4.799^***^	4.948^***^	5.069^***^	5.171^***^	5.425^***^	1.509^***^
	(9.665)	(10.340)	(10.577)	(10.916)	(11.177)	(3.988)
*N*	337	337	337	337	337	337
*Adjusted R* ^2^	0.123	0.148	0.086	0.096	0.101	0.564
*F*	8.382	7.930	6.047	5.201	5.845	46.054

*N* = 337, ^*^p < 0.1, ^**^p < 0.05; ^***^p < 0.01.

The results support the hypotheses about the effect of perceived risk on purchase intention, and moderation of consumer knowledge on the relationship between perceived risk and consumer trust. To further test the mediation effect of consumer trust and moderated path analysis (Edwards and Lambert, 2007), we utilize the SEM model to verify the influence path. Next, we examined the moderation effect of consumer knowledge on the mediation path.

### Analysis of mediation effect

To further test the mediation effect and moderation effect, we utilized the Structural equation modeling (SEM) method to test the influence path between consumers’ perceived risk, quality trust, green trust, and purchase intention. Structural equation modeling is a helpful tool and method for exploring the associations between latent variables, which is in line with the theory about the interrelationships among the variables (Chin, 1998). Structural equation modeling consists of the measurement model and the structural model (Anderson and Gerbing, 1988). The measurement model explores the associations between measurement items and latent variables. The structural model mainly focuses on exploring the latent variables’ associations. Next, we further test the mediating effect of quality trust and green trust, and the moderating effect of consumer knowledge. We included all the control variables in the following analyses on the mediation effect and moderation effect.

The structural equation path coefficients are shown in Table 5. Consumer perceived risk negatively affects quality trust (*β* = −0.192, *p* < 0.01) and green trust (*β* = −0.151, *p* < 0.05), which supports H2a and H2b. Meanwhile, both quality trust (*β* = 0.699, *p* < 0.01) and green trust (*β* = 0.183, *p* < 0.05) positively influence purchase intention, which confirms H3a and H3b.

**Table 5:** Structural equation path coefficients.

Hypothetical path	Standardized coefficient	Std. Err.	*p* > |z|	95% C.I.		Conclusion
				Lower	Upper	
H2a Perceived Risk - > quality trust	−0.192	0.069	0.005	−0.328	−0.057	Support
H2b Perceived Risk - > green trust	−0.151	0.069	0.029	−0.287	−0.016	Support
H3a Quality Trust - > purchase intention	0.699	0.076	0.000	0.550	0.848	Support
H3b Green Trust - > purchase intention	0.183	0.080	0.023	0.026	0.341	Support

We employed SPSS PROCESS macro of Hayes (2017) to further examine the mediating effect of quality trust and green trust in the influence of perceived risk on purchase intention. The results in Table 6 showed that the overall effect of perceived risk on purchase intention was significant (*β* = −0.120, 95% CI [−0.191, −0.050]), which supports H1. The mediating effect of quality trust (*β* = −0.086, 95% CI [−0.142, −0.036]) and green trust (*β* = −0.035, 95% CI [−0.067, −0.007]) were both significant, which support hypotheses H4a and H4b. In addition, the direct effect of perceived risk on purchase intention is not significant when both quality trust and green trust are present (*β* = 0.004, 95% CI [−0.093, 0.101]). Thus, the two types of consumer trust fully mediate the effect of perceived risk on purchase intention.

**Table 6:** Mediating effect analysis of quality trust and green trust.

Hypothetical path	Total Effect	Direct Effect	Indirect Effect	95% C.I.		Conclusion
				Lower	Upper	
H1 Perceived risk— > purchase intention	−0.120			−0.191	−0.050	Support
		0.004		−0.093	0.101	
H4a Perceived risk— > quality trust > purchase intention			−0.086	−0.142	−0.036	Support
H4b Perceived risk— > green trust > purchase intention			−0.035	−0.067	−0.007	Support

### Analysis of moderating effect

Next, we analyzed the difference in perceived risk’s influence on quality trust under different levels of consumer knowledge (Table 7).

**Table 7:** Analysis of the moderating effect of consumer knowledge.

Moderating effect model	Standardized coefficient	Std. Err.	95% C.I.		Conclusion
			Lower	Upper	
H5a Perceived risk— > quality trust	−0.121	0.036	−0.191	−0.050	Support
−1 SD (consumer knowledge)	−0.365	0.070	−0.502	−0.228	
Mean (consumer knowledge)	−0.221	0.048	−0.316	−0.126	
+1 SD (consumer knowledge)	−0.077	0.060	−0.195	0.041	
H6a The moderated mediating effect of quality trust	0.066	0.020	0.028	0.107	Support
H5b Perceived Risk— > green trust					Support
−1 SD (consumer knowledge)	−0.192	0.059	−0.307	−0.076	
Mean (consumer knowledge)	−0.108	0.040	−0.187	−0.028	
+1 SD (consumer knowledge)	−0.023	0.049	−0.121	0.074	
H6b The moderated mediating effect of green trust	0.039	0.017	0.006	0.074	Support

The results show that when consumer knowledge was at a lower level (−1 SD), perceived risk had a stronger negative impact on quality trust (*β* = −0.365, 95% CI [−0.502, −0.228]). When consumer knowledge is average, the negative impact of perceived risk on quality trust is weak (*β* = −0.221, 95% CI [−0.316, −0.126]). When consumer knowledge is at a higher level (+ 1 SD), perceived risk’s influence on quality trust was not significant (*β* = −0.077, 95% CI [−0.195, 0.041]). The results supported H5a, that is, the conditional effect of perceived risk on quality trust are significantly stronger for consumer knowledge at low levels. High consumer knowledge suppresses the negative correlation between perceived risk and trust, making that relationship non-significant. In addition, the moderating effect of consumer knowledge on the mediating effect of quality trust was also confirmed (*β* = 0.066, 95% CI [0.028, 0.107]); hence hypothesis H6a was supported.

We also analyzed the difference in perceived risk’s influence on green trust under different levels of consumer knowledge. Table 7 shows that when consumer knowledge was at a lower level (−1 SD), perceived risk had a stronger negative impact on green trust (*β* = −0.192, 95% CI [−0.307, −0.076]). When consumer knowledge is average, the negative impact of perceived risk on quality trust is weak (*β* = −0.108, 95% CI [−0.187, −0.028]). When consumer knowledge is at a higher level (+ 1 SD), perceived risk’s influence on quality trust was not significant (*β* = −0.023, 95% CI [−0.121, 0.074]). The results supported H5b, that is, consumer knowledge of water-saving appliances can alleviate the negative impact of perceived risk on green trust, and high levels of consumer knowledge can make the negative effect of perceived risk on trust non-significant. In addition, the moderating effect of consumer knowledge on the mediating effect of green trust was also confirmed (*β* = 0.039, 95% CI [0.006, 0.074]); hence hypothesis H6b was supported.

**Figure 2:**
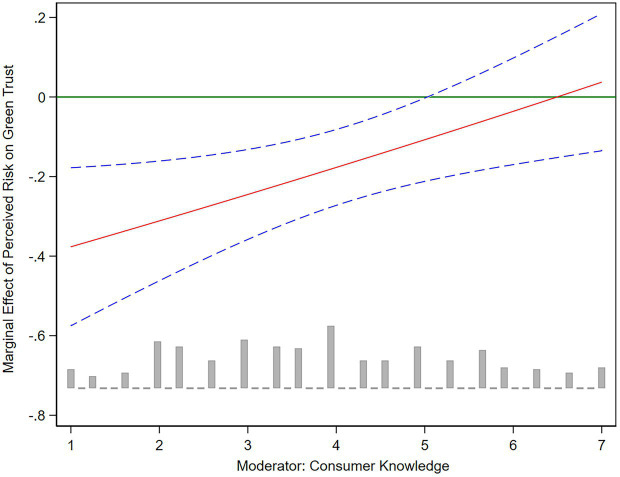
The marginal effect of perceived risk on quality trust.

**Figure 3:**
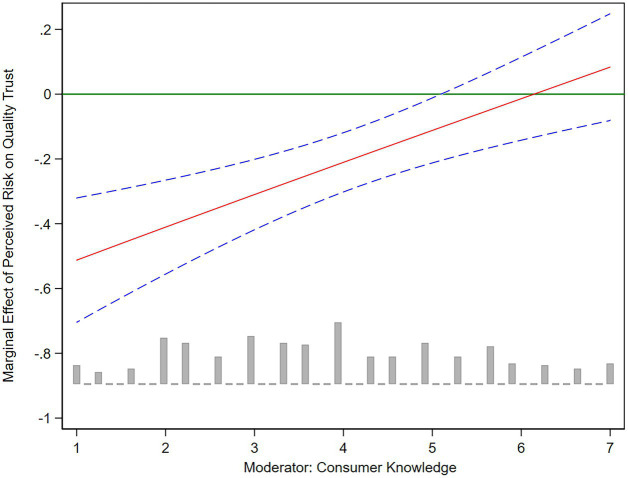
The marginal effect of perceived risk on green trust.

We further plotted the marginal effect of perceived risk affecting quality trust and green trust under the moderation of consumer knowledge. As shown in Figures 2, 3, the solid red line represents the influence coefficient of perceived risk on quality trust and green trust, and the blue dotted line represents the 95% confidence interval. For consumers with higher levels of product knowledge, their perceived risk showed a weaker negative influence on quality trust and green trust. Specifically, when the consumer knowledge enters the high level (CK ≥ 5), the negative impacts of perceived risk on quality trust and green trust turn to not significant. Therefore, for consumers with sufficient product knowledge about water-saving appliances, their trust level in water-saving appliances would be less sensitive to perceived risk.

## Discussion and implications

### Discussion

The popularization of water-use appliances is the main force that raises the water demand (Wang et al., 2014; Vieira et al., 2017). Encouraging residents to adopt water-saving appliances has been considered a critical way to conserve water resources. In promoting consumer purchase intention on water-saving appliances, conventional wisdom mainly focuses on external factors, such as demographic characteristics and product characteristics (Mu et al., 2014; Tapsuwan et al., 2018; Fan et al., 2019). In contrast to the previous literature, we draw on the risk perception perspective and examine the psychological mechanisms that influence purchase intention. By constructing a moderated dual-mediation model, we revealed the influence of consumers’ perceived risk on purchase intention of water-saving appliances and the mediating effect of consumer trust (*quality trust* and *green trust*), and the moderating effect of consumer knowledge. The main conclusions are as follows.

In purchasing water-saving appliances, consumers’ perceived risk consists of functional risk, economic risk, and psychological risk, which negatively impact consumers’ purchase intention of water-saving appliances. We found that perceived risk about water-saving appliances’ functional quality, user experience, maintenance costs, and psychological anxiety are major factors that inhibit consumers’ willingness to purchase.

Consumers’ quality trust and green trust in water-saving appliances play negative mediating roles between perceived risk and purchase intention. The mediating effect of quality trust is stronger than that of green trust. As analysis results present, consumers who perceive risks show lower quality and green trust in water-saving appliances, indirectly affecting their willingness to buy them. Meanwhile, compared with green trust, consumers’ perceived risk has a stronger negative impact on quality trust, and quality trust has a stronger positive effect on purchase intention.

Consumer knowledge can mitigate the negative impact of perceived risk on consumer trust and the indirect effect of consumer trust on purchase intention. We found that for consumers with more affluent knowledge of water-saving appliances, their perceived risk had a lower level of negative impact on quality trust and green trust. For this segment of consumers, the indirect effect of perceived risk on purchase intention is weaker.

### Theoretical implication

In the field of green products, water-saving appliances are essential in conserving our limited water resources, especially in those water-scarcity areas. To further the research on consumer purchase intention of water-saving appliances, we draw on perceived risk theory and examine the influence mechanism of perceived risk on purchase intention. We constructed a moderated dual-mediation model by introducing quality and green trust as the mediators, and consumer knowledge as the moderator, thereby revealing how and when perceived risk shapes purchase intention. Based on the above findings, our contribution is threefold.

Compared with previous studies focusing on the impact of education, and income, we revealed the psychological factors influencing purchase intention on water-saving appliances. Conventional wisdom primarily focuses on consumer demographic such as education and income (Martínez-Espiñeira and García-Valiñas, 2013; Tapsuwan et al., 2018), and product characteristics such as water-saving performance (Hustvedt et al., 2013; Fan et al., 2019), leaving the psychological factors and mechanisms unresolved. Drawing on perceived risk theory, we explored the multiple psychological risks that influence purchase intention, including function risk, economic risk, and psychological risk, thereby extending the micro-foundation of how to promote consumer purchase intention on water-saving appliances.

Perceived risk has long been identified as harming green purchase intention (Zhuang et al., 2021). In terms of how perceived risk inhibits the purchase intention of green products, previous wisdom mainly sheds light on the mediation effect of green trust (Chen and Chang, 2012; Juliana et al., 2020). Meanwhile, the dual mediation effect of green trust and quality trust has yet to receive attention. By integrating quality trust and green trust into the same model, our study is one of the first to examine the dual mediation effect of quality trust and green trust, which advanced in theorizing the trust mechanism between perceived risk and purchase intention of green products.

Lastly, our study is one of the first to examine the interaction of perceived risk and consumer knowledge on consumer trust and purchaser intention of green products such as water-saving appliances. A recent meta-analysis research has suggested that perceived risk negatively influences the purchase intention of green products (Zhuang et al., 2021). However, little research has explored consumer knowledge as the moderator of the effects of perceived risk (Juliana et al., 2020). This shortcoming is vital because consumer knowledge largely determines the degree to which perception factors shape trust and purchase intention (Lee et al., 2015). We theorized and empirically tested consumer knowledge as the moderator and examined the contingent influence of perceived risk on the purchase intention of water-saving appliances, and the dual mediating effect of green trust and quality trust. In doing so, we enrich the perceived risk literature by framing consumer knowledge as a contextual contingency factor for the influence of perceived risks on green purchase intention.

### Managerial implication

With the climbing tensions in water supply worldwide, water-saving appliances have been considered vital for water conservation (Fan et al., 2019). In practical terms, enterprises must minimize consumers’ perceived risk regarding green product purchases (Zhuang et al., 2021). Therefore, our research on how and when perceived risk negatively influences purchase intention could offer several practical implications.

First, our findings indicate that the purchase intention of water-saving appliances is negatively associated with perceived risks, including function risk, economic risk, and psychological risk. Therefore, the government and enterprises should take action to minimize those perceived risks regarding water-saving appliances. The government should strengthen the support and supervision for after-sales services of water-saving appliances. Consumers’ perceived risk mainly comes from uncertainty about the post-purchase experience (Marriott and Williams, 2018). In this regard, government departments like quality supervision, industry and commerce, and water affairs can supervise merchants to improve after-sales services. For instance, merchants could extend the period of unconditional return, provide free on-site maintenance, timely online answering, etc. The government could issue certificates to merchants with excellent after-sales service and publicize their excellent practices, by doing this to guide the continuous standardization and improvement of after-sales service in the water-saving appliance market, eliminating consumers’ anxieties about purchasing water-saving appliances as much as possible.

Moreover, our findings suggest that consumers’ perceived risk could negatively influence quality and green trust, thus depressing their purchase intention. To block the negative influence of perceived risk on purchase intention, the government and firms should enhance consumers’ trust in water-saving appliances. When consumers buy water-saving appliances, besides their expectations for elementary product quality, they also have specific requirements for water-saving performance (Fan et al., 2019). Therefore, in the design and production of water-saving appliances, the product’s functional quality and water-saving performance should be considered. Governments and firms should improve national and industry standards for water-saving appliances, requiring water appliances with higher cleaning performance and water efficiency. On this basis, governments should enact policies and regulations and operate joint enforcement, ensuring that only qualified water-saving appliances can enter the sales market, and retire unqualified products as soon as possible.

Finally, we also found that after perceived risks, consumers with higher levels of knowledge are less likely to reduce their trust, and in turn purchase intention of water-saving appliances. What a consumer knows about a product or service is crucial to how it is marketed, and this is particularly true in the case of information-intensive products (Vigar-Ellis et al., 2015). Considering this, the government and merchants could collaborate to strengthen public advertising, thus popularizing the knowledge about water-saving appliances. Besides, the government could promote water-saving appliances *via* public procurement, especially in those public places, thus helping more residents learn and understand water-saving appliances.

## Limitations and future research

As with all studies, though the present manuscript has some interesting findings and implications, it is not without some inherent limitations. First, this study relies on cross-sectional data and self-reported data. Thus, we cannot flatly claim the causality demonstrated in our model since we did not employ a rigorous longitudinal design. Future research can adopt longitudinal, experimental, or qualitative designs to replicate and extend our study’s findings.

Second, the sample of this research is relatively small and needs to be fully representative of the Chinese population. For instance, our sample is unbalanced, with more females participating. Future research may replicate our study with equal female and male participants. Besides, while the residents’ water conservation awareness varies across regions, 61.42% of our respondents come from Beijing, Guangdong, and Jiangsu, the developed regions in China. Although we controlled the gender, education, age, occupation, location, and income in regression analyses, caution still needs to be taken when generalizing the research findings. Future studies can further validate our findings based on more extensive and diverse population strata samples.

Third, like prior literature on consumer perceived risk (Park et al., 2016; Marriott and Williams, 2018), we evaluated the perceived risk of purchasing water-saving appliances at a specific time point. Since perceived risk’s facets and influence might vary for different product categories and decision-making phases, this shortcoming could be vital in marketing water-saving appliances. To accurately capture the change of perceived risk as well as the level of perceived risk, an experience sampling methodology may be adopted to assess the trajectories of perceived risk and then examine its facets and influences in future research.

## Data availability statement

The raw data supporting the conclusions of this article will be made available by the authors, without undue reservation.

## Ethics statement

All procedures performed in studies involving human participants were in accordance with the ethical standards of the institutional and/or national research committee and with the 1964 Helsinki declaration and its later amendments or comparable ethical standards.

## Author contributions

WT and TM: conceptualization. WT: methodology, software, formal analysis, data curation, writing—original draft preparation, and funding acquisition. TM: validation, writing—review and editing. All authors contributed to the article and approved the submitted version.

## Funding

This study was supported by Fundamental Research Funds for the Central Universities B220201057.

## Conflict of interest

The authors declare that the research was conducted in the absence of any commercial or financial relationships that could be construed as a potential conflict of interest.

## Publisher’s note

All claims expressed in this article are solely those of the authors and do not necessarily represent those of their affiliated organizations, or those of the publisher, the editors and the reviewers. Any product that may be evaluated in this article, or claim that may be made by its manufacturer, is not guaranteed or endorsed by the publisher.
